# Identifying traumatization in young children through structured play: validation of the Odense Child Trauma Screening (OCTS) in Lithuania

**DOI:** 10.1080/20008066.2025.2474373

**Published:** 2025-03-10

**Authors:** Paulina Zelviene, Odeta Gelezelyte, Agniete Kairyte, Ask Elklit, Sille Schandorph Løkkegaard, Evaldas Kazlauskas

**Affiliations:** a Center for Psychotraumatology, Institute of Psychology, Vilnius University, Vilnius, Lithuania; b Department of Psychology, National Center of Psychotraumatology, University of Southern Denmark, Odense, Denmark

**Keywords:** Story stem test, validity, traumatization, PTSD, young children, Story stem test, validez, traumatización, trastorno de estrés postraumático (TEPT), niños pequeños

## Abstract

**Background:** There is a need for valid methods to evaluate young children’s (4–8 years) psychological difficulties related to traumatic experiences. The Odense Child Trauma Screening (OCTS), developed by Danish researchers, is a play-based story stem assessment tool developed to screen for indicators of traumatization in young children. Just a few studies of the OCTS have been published so far.

**Objective:** The current study aimed to test the reliability and convergent validity of the OCTS in the Lithuanian community and risk subsamples of young children aged 4–8 years.

**Method:** The total sample consisted of 209 participants (58.9% girls) from the community (47.4%) and risk (52.6%) subsamples, *M*age = 6.29 (*SD *= 1.48). All children were screened with the OCTS, and caregivers completed self-report questionnaires: demographics, the Child and Adolescent Trauma Screen-Caregiver (CATS-C), and the Strengths and Difficulties Questionnaire (SDQ).

**Results:** The data suggests that the OCTS has good inter-rater reliability. The OCTS, SDQ, and CATS-C scores were significantly higher in the risk subsample, with small to large effect sizes. Boys and younger children (3–4-year-olds) scored higher on the OCTS. Out of all the OCTS stories, the Burnt hand story had significant correlation coefficients with all the CATS-C PTSD symptoms.

**Conclusions:** The study provides initial information about the reliability and the validity of the OCTS and calls for further exploration of this instrument. There were also variations in scores between the Lithuanian data and an earlier study of the Danish sample. Future studies on the OCTS would benefit from further cross-cultural, reliability and the validity examination.

## HIGHLIGHTS

- This is the first empirical study that evaluated the reliability and convergent validity of the newly developed the Odense Child Trauma Screening (OCTS) measure, targeting to identify possible representational signs of young children’s (aged 4–8 years) traumatization outside Denmark.
- The study supports the inter-rater reliability of the OCTS and informs that the OCTS administration and coding procedures can be applied in various settings.
- The study provides initial information about the reliability and the validity of the OCTS outside its country of origin and calls for further exploration of this instrument.
- Considering the lack of age-appropriate screening tools for young children’s traumatization, this paper provides important data for research and clinical practice about the novel measure much needed for specialists working with child abuse, neglect, and trauma.

## Abstract (es)

**Antecedentes:** Existe una necesidad de métodos válidos para evaluar las dificultades psicológicas de los niños pequeños (de 4 a 8 años) relacionadas con experiencias traumáticas. El Odense Child Trauma Screening (OCTS), desarrollado por investigadores daneses, es una herramienta de evaluación de historias basada en el juego desarrollada para detectar indicadores de traumatización en niños pequeños. Hasta el momento sólo se han publicado unos pocos estudios sobre el OCTS.

**Objetivo:** El presente estudio tuvo como objetivo probar la confiabilidad y validez convergente de la OCTS en la comunidad lituana y en submuestras de riesgo de niños pequeños de 4 a 8 años.

**Método:** La muestra total estuvo compuesta por 209 participantes (58,9% niñas) de las submuestras comunitaria (47,4%) y de riesgo (52,6%), *M*_(edad)_ = 6,29 (*DE* = 1,48). Todos los niños fueron evaluados con la OCTS, y los cuidadores completaron cuestionarios de autoinforme: datos demográficos, el Cuestionario de Detección de Trauma Infantil y Adolescente para Cuidadores (CATS-C en sus siglas en ingles) y el Cuestionario de Fortalezas y Dificultades (SDQ en sus siglas en ingles).

**Resultados:** Los datos sugieren que la OCTS tiene una buena confiabilidad entre evaluadores. Las puntuaciones de OCTS, SDQ y CATS-C fueron significativamente más altas en la submuestra de riesgo, con tamaños de efecto de pequeños a grandes. Los niños y los niños más pequeños (3-4 años) obtuvieron puntuaciones más altas en la OCTS. De todas las historias de OCTS, la historia de la mano quemada tuvo coeficientes de correlación significativos con todos los síntomas de TEPT de CATS-C.

**Conclusiones:** El estudio proporciona información inicial sobre la fiabilidad y validez de la OCTS y exige una mayor exploración de este instrumento. También hubo variaciones en las puntuaciones entre los datos lituanos y un estudio anterior de la muestra danesa. Los estudios futuros sobre la OCTS se beneficiarían de un mayor examen intercultural, de confiabilidad y validez.

**Abbreviations:** CATS-C: Child and Adolescent Trauma Screen Caregiver version; DIPA: Diagnostic Infant and Preschool Assessment; ICC: intraclass correlation coefficient; OCTS: Odense Child Trauma Screening; PTEs: potentially traumatic events; PTSD: posttraumatic stress disorder; SDQ: Strengths and Difficulties Questionnaire

## 1. Introduction

The recognition of young children’s trauma-related difficulties is challenging (American Psychiatric Association, 2022; World Health Organization, 2024). Most commonly, caregiver reports or multi-informant approaches are used for the evaluation of a child’s trauma-related symptoms (De Los Reyes et al., 2015; Woolgar et al., 2022). However, there is a high need to enable young children as valid informants about their trauma-related mental health for a variety of reasons. One of them is that due to the complexity of trauma-related symptoms, it can be difficult for caregivers to notice and assess them (de Young et al., 2011). Also, child trauma and abuse cause strong feelings in caretakers like shock, guilt, shame, and denial (Holt et al., 2014) that may impede report and relevant care. Research shows that caregivers might underreport internalized problems compared to child reports when children are younger (De Los Reyes et al., 2015; Nader, 2007). Also, the caregivers may be unavailable, or they are unreliable informants where trauma-focused care is needed, e.g. in institutions providing support to child victims of violence (Løkkegaard et al., 2024) or in foster care systems. The last but no less important reason is to recognize a human rights perspective on children as persons with juridical rights to be protected, heard, and included (United Nations, 1989).

Due to their developmental capacities, younger children may find it challenging to articulate their emotions and experiences related to traumatic events. They might not yet have the language skills or psychological awareness necessary to accurately express their inner states (Salmon & Bryant, 2002). Young children play; therefore, play-based assessment tools can be effective for an in-depth examination of the inner world of children who are at risk for traumatization (Løkkegaard et al., 2021). The Odense Child Trauma Screening (OCTS, Løkkegaard et al., 2017, 2018) is one of such play-based measures that has been developed to screen for indicators of traumatization in young children based on how they behave and play within a structured play setting that induces a controlled degree of arousal and distress (Alkærsig et al., 2024). The OCTS employs a story stem approach involving common child-related themes and a play with a LEGO® house and dolls. Story stems are constructed to elicit responses that can be indicative of traumatization, and playing allows them to project their feelings, experiences, and cognitive schemas into the narrative (Løkkegaard et al., 2017).

A few studies on the validity and reliability of the OCTS have been published so far. For the initial validation of the OCTS, in a sample of 49 Danish children aged 4.5–8.9 years from risk and community samples, the internal consistency and inter-rater reliability were excellent for the total OCTS score, and each story’s partial scores, the test-retest reliability was acceptable (Løkkegaard et al., 2021). Moreover, this initial study supported the convergent validity of the OCTS with moderate and significant correlations between the OCTS total score and the PTSD, major depressive disorder, and reactive attachment disorder (RAD) scales from the Diagnostic Infant and Preschool Assessment (DIPA) (Løkkegaard et al., 2019), as well as the Strengths and Difficulties Questionnaire (SDQ) (Goodman, 1997) total difficulties. Furthermore, the OCTS showed promising results as a screening tool for signs of traumatization (Løkkegaard et al., 2021). Further study in a large sample of 169 children aged 4–8 from the general population provided preliminary Danish norms of the OCTS (Alkærsig et al., 2024). It was found that in a few of the OCTS code scores, boys had higher scores than girls. Importantly, it was also found that the OCTS total score and three of the partial scores (score per story stem) had a tendency to decrease with older age (Alkærsig et al., 2024). The associations between the OCTS, SDQ, and DIPA trauma list scores provided more convergent validity for the Danish version of the OCTS (Alkærsig et al., 2024).

Until recently, all available data on the psychometric properties of the OCTS were available only in Danish samples. The first empirical data on the reliability and validity of the OCTS outside Denmark originated in Lithuania, revealing promising findings. A pilot study in a sample of 52 Lithuanian children from risk and community subsamples, aged 3–9 years, supported internal consistency and inter-rater reliability. It showed significant moderate correlations between the total score of the OCTS and the SDQ external difficulties: Conduct problems and Hyperactivity (Zelviene et al., 2024). As the OCTS is a new measure, more data is needed about the psychometric properties in the community and the risk for traumatization samples of young children, especially outside Denmark.

While story stem measures offer valuable insights into a child’s mental representations of themselves and others, as well as their emotion regulation strategies, which are crucial for individual case formulation (Tang et al., 2018), it is also essential to examine the validity of story stem measures compared to other measures of child symptomatology and difficulties, age and gender differences and reliability. Therefore, the present study sought to assess the reliability and convergent validity of the OCTS in a sample of children younger than eight years old from community and risk subsamples. The focus was on evaluating the OCTS’s internal consistency and inter-rater reliability. For the convergent validity analyses, the aim was to explore the OCTS scores in relation to the child’s internalizing and externalizing problems using the SDQ (Goodman, 1997) caregiver’s version and DSM-5 PTSD symptoms using the Child and Adolescent Trauma Screen-Caregiver’s (CATS-C) version (Sachser et al., 2017). The study further investigated the OCTS’s capability to differentiate between those at risk for PTSD and those without such risk. Furthermore, the study aimed to assess the OCTS scores among boys and girls as well as across different age groups.

## 2. Methods

### 2.1. Procedure and participants

The total sample comprised community and risk subsamples; therefore, the participants were recruited from various educational, psychological, and social support institutions in Lithuania.

The study was carried out in seven public and private kindergartens and schools for the community subsample. The teachers distributed study information and informed consent forms for the caregivers across groups of children whose age was appropriate for the study. Caregivers who agreed and signed the informed consent received the invitations for the self-report survey on an online platform. Children whose caregivers filled out the online survey were invited and provided their own consent to participate in the OCTS, which was carried out in kindergartens or schools. The OCTS interviewers for the community subsample were nine specialists – master’s students in clinical psychology and clinical psychologists; all had participated in the OCTS administration training and supervisions, provided by the Danish authors of the OCTS throughout the study.

For the risk subsample, data was collected in collaboration with 12 clinical psychologists from six organizations supporting and working with potential child victims of sexual abuse and providing support for children from risk and foster families. All psychologists participated in the OCTS administration training and supervisions, provided by the Danish team of the OCTS throughout the study. Informed consent and information about the survey were distributed by the psychologists within the institutions to the legal guardians or caregivers of children. After obtaining signed informed consent, caregivers participated in the self-report survey within the institution. Only those children whose caregivers or legal guardians filled out the survey participated in the OCTS administered within the institution.

In total, video data for 221 children was collected. However, the data of eight participants were excluded from the study because of an incomplete OCTS (i.e. less than four stories were played or a significant number of codes were not possible to code), two children were older than nine years, and there were two children whose age was unknown. In the sample, there were two 3-year-old children and nine 9-year-old children, while the recommended age for administering the OCTS is from 4 to 8 years (Løkkegaard et al., 2017). However, after evaluating that the OCTS scores of those children were not considered outliers, we decided to include their data in the final analyses.

Eventually, the final dataset consisted of 209 participants who were divided into two groups. The community subsample comprised children interviewed in kindergartens and schools, and the risk subsample comprised children interviewed in institutions providing support for children from risk families and in foster care institutions. There were 99 (47.4%) children from the community subsample, and the remaining 110 (52.6%) were defined as a risk subsample. The total sample comprised 123 (58.9%) girls and 86 (41.1%) boys. The average age was 6.29 (*SD *= 1.48) years. The sample demographic information and comparisons between the study subsamples can be found in Table 1. On average, children from the risk subsample were older (*M* = 6.78, *SD* = 1.44) than those from the community subsample (*M* = 5.74, *SD* = 1.32; *t*(207) = 5.44, *p* < .001). Various risk factors related to the child’s current living situation, caregiver’s education, employment status, alcohol or drug misuse, imprisonment experience, and the child’s usage of professional psychological help were more prevalent among the children in the risk subgroup.

**Table 1.** Sample demographic characteristics and comparisons between study subsamples.

Demographic characteristics	Full sample (*N* = 209)		Community subsample (*n *= 99)		Risk subsample (*n* = 110)		*χ^2^* (*df*)*/t (df)*	*p*	Φ/Cramer’s V/Cohen’s *d*
	*n*/*M* (*SD*)	%	*n*/*M* (*SD*)	%	*n*/*M* (*SD*)	%			
Sex									
Girls	123	58.9	58	58.6	65	59.1	.005 (1)	.941	0.005
Boys	86	41.1	41	41.4	45	40.9			
Age	6.29 (1.48)		5.74 (1.32)		6.78 (1.44)		5.44 (207)	**<.001**	0.753
3 years	2	1.0	2	2.0	0	0	32.43 (6)	**<**.**001**	0.394
4 years	23	11.0	13	13.1	10	9.1			
5 years	47	22.5	34	34.3	13	11.8			
6 years	47	22.5	26	26.3	21	19.1			
7 years	30	14.4	9	9.1	21	19.1			
8 years	51	24.4	14	14.1	37	33.6			
9 years	9	4.3	1	1.0	8	7.3			
Siblings									
Yes	162	77.5	75	75.8	87	79.1	0.33 (1)	.564	0.040
No	47	22.5	24	24.2	23	20.9			
Currently lives									
with both parents	118	56.5	89	89.9	29	26.4	90.54 (5)	**<**.**001**	0.658
alternately with mother and father	2	1.0	1	1.0	1	0.9			
with one parent	44	21.1	9	9.1	35	31.8			
with other relatives	6	2.9	0	0	6	5.5			
with guardians	32	15.3	0	0	32	29.1			
in the institution	7	3.3	0	0	7	6.4			
High education have/has (*N* = 204)									
Both caregivers	87	42.6	61	61.6	26	24.8	36.67 (2)	**<**.**001**	0.424
One caregiver	53	26.0	25	25.3	28	26.7			
Neither of caregivers	64	31.4	13	13.1	51	48.6			
Current employment situation (*N* = 206)									
Both caregivers work	115	55.8	77	77.8	38	35.5	40.80 (2)	**<**.**001**	0.445
One caregiver works	78	37.9	22	22.2	56	52.3			
Neither of caregivers work	13	6.3	0	0.0	13	12.1			
Alcohol or drug misuse by caregivers (*N* = 207)									
No	176	85.0	96	97.0	80	74.1		**<**.001*	
Yes	31	15.0	3	3.0	28	25.9			
Mental illness of caregivers (*N* = 207)									
No	197	95.2	96	97.0	101	93.5		.336*	
Yes	10	4.8	3	3.0	7	6.5			
Any of the caregivers did time in prison (*N* = 207)									
No	197	95.2	99	100.0	98	90.7		.002*	
Yes	10	4.8	0	0.0	10	9.3			
Child has received professional psychological services (*N* = 208)									
No	145	69.7	91	91.9	54	49.5	44.13	**<**.**001**	0.461
Yes	63	30.3	8	8.1	55	50.5			

Note*.* Significant differences are presented in bold. *Fisher’s exact test.

### 2.2. Measures

Sociodemographic data was received from the caregivers. Data was collected on the child’s sex, age, siblings, living situation, and whether or not the child received professional psychological help. Also included was information on caregivers’ education, employment status, alcohol or drug misuse, mental illness, and history of imprisonment.

*The Odense Child Trauma Screening (OCTS)* (Løkkegaard et al., 2021) is a story stem screening tool for recognizing signs of traumatization, recommended for use in children aged 4–8 years. The OCTS is designed to use storytelling and playing with figures in a structured play interview. The tool includes five main story stems – one warm-up baseline Birthday story and four conflict stories about Biking, Nightmare, Burnt hand, and Stomach ache. Stories are played using the LEGO® house and dolls that represent family figures. All story stems are built up until the most dramatic point (e.g. the Burnt hand story begins with the family preparing for dinner, when a child representative figure at some point burns a hand on a pan). Then, the child being assessed is asked to continue the story by telling and showing what happens next using the LEGO® figures. The methodology has an additional optional Animal story with animal figures (e.g. a group of giraffes or a crocodile), which can be used if the play with family figures and house does not provide sufficient material for reliable coding. In this study, an optional Animal story was completed in almost half of the evaluations (47.9%, *n* = 100) to have enough data for statistical analyses.

The OCTS assessment is administered by a trained psychologist and videotaped for subsequent coding. The administration of the OCTS takes 20–30 minutes and follows the instructions provided in the administration manual (Løkkegaard et al., 2017). If the Animal story is added, administration time is usually about 5–10 minutes longer. All played conflict stories are coded using the OCTS coding manual (Løkkegaard et al., 2018). At least four stories should be administered for a valid evaluation and calculation of a total score. The OCTS administration manual and coding manuals were translated into Lithuanian. The pilot study results published by Zelviene et al. (2024) revealed the preliminary OCTS psychometric characteristics in the Lithuanian sample. Reliability analysis indicated acceptable internal consistency, with Cronbach’s α coefficients for the total OCTS and separate stories varying from .75 to .90. The agreement between different raters of the OCTS videos also demonstrated good inter-rater reliability, with intraclass correlation coefficients ranging from .82 to .89 (Zelviene et al., 2024).

The OCTS narrative coding system has 27 codes, which are divided into five categories: (1) Child engagement and narrative production (codes 1–4), (2) Nature of the narrative (codes 5–6), (3) Adult representations in the narrative (codes 7–12), (4) Child representations in the narrative (codes 13–21), and (5) Disorganized phenomena (codes 22–27). The codes within the four latter categories were based on previous studies of children exposed to various traumatic experiences that used story stem tools (Løkkegaard et al., 2021). Child engagement and narrative production category codes (1–4) are rated with dichotomous scores (0 = phenomenon is not present; 2 = phenomenon present). This category is used only to make an assessment of the child in the interview situation and to clarify if the administration and child narrative production is adequate to conduct a reliable rating of the following codes 5–27, for this reason, a categorical distinction of the behaviour being present or not is necessary, and the scores of this category (codes 1–4) are excluded from the total scores of the separate stories and from the total score of the OCTS. The scoring of the OCTS has four steps. First, the raw scores for codes 5–27, are rated on a three-point scale (0 = phenomenon is not present; 1 = phenomenon expressed less clearly; 2 = phenomenon definitely present). All codes have specific descriptions and clear guidance on what situation each score should be assigned (Løkkegaard et al., 2018). Second, the raw scores of all codes are converted into weighted scores according to the red (codes 9–11, 14–16, 18, 20, 22–27), yellow (codes 4, 5, 7, 8, 12, 13, 17, 19, 21) and green (codes 1–3, 6) colours. The red codes represent the child narratives that are highly likely to indicate traumatization, e.g. sexual material in behaviour (the raw scores of 1 and 2 are weighted into 1 for the red codes), the yellow codes represent characteristics or themes that are associated with possible traumatization or another vulnerability, e.g. adult is controlling (a raw score of 1 is weighted into 0, and a raw score of 2 is weighted into 1 for the yellow codes), and green codes, that are not directly related to traumatization but are included to describe general child engagement and compliance with the screening situation, e.g. engagement in the story (the raw scores of 1 and 2 are weighted into 0). The differential weighing is based on empirical evidence indicating that certain representations are predominantly associated with traumatization, while others are also observed in children exhibiting symptomatic behaviours, such as mood or behavioural disorders (Løkkegaard et al., 2017; Løkkegaard et al., 2018; Løkkegaard et al., 2021). The third step, the partial score of each story is calculated by adding the weighted scores. The partial score of a story may range from 0 to 23. The last step, the total score of the OCTS is calculated by summing up of all stories’ weighted scores and dividing by the number of the stories played during the interview, with higher scores indicating a higher probability of experienced traumatization (Løkkegaard et al., 2018).

The OCTS administration procedure for community and risk subsamples occurred within the institutions where the child was and according to the OCTS administration manual (Løkkegaard et al., 2017). The OCTS interviews took place in a separate room (e.g. a psychologist’s office). The interviewer prepared a LEGO® house and figures for the OCTS test, set up a video camera in advance, invited the child to take part in testing in the age-appropriate language of the child, and then, after the child’s consent, walked the child into the room. The interviewer presented the OCTS test and procedure and informed the child that the play would be videotaped. The psychologist also monitored the child’s engagement, and if the child did not want to play, the OCTS interview was stopped. On average, the OCTS interview took from 30 to 40 min. to complete. Very often, in the community subsample, the children who had already taken part in the OCTS would tell the other children in the group about their experiences. Hence, the children were interested in advance and eagerly waited for the invitation.

*The Strengths and Difficulties Questionnaire (SDQ)* (Goodman, 1997) is widely used for a child’s internalizing and externalizing problems screening. This study used the caregiver’s version of the SDQ for 4–17-year-olds. The SDQ comprises a Prosocial behaviour subscale that measures a child’s strengths and four subscales that measure a child’s difficulties – Conduct problems and Hyperactivity, representing external difficulties, and Emotional problems and Peer problems, representing internal difficulties, with five items per subscale. Caregivers were asked to think about their child’s behaviour over the last six months and evaluate each item on a three-point scale (0 = Not true; 2 = Certainly true). The total scores of each subscale may range from 0 to 10, with higher scores on the Prosocial behaviour scale indicating better functioning and higher scores on the rest of the subscales representing more significant difficulties of a corresponding subscale. The internal difficulties were calculated by summing items of Emotional problems and Peer problems subscales, and external difficulties were measured by summing Conduct problems and Hyperactivity subscales (Goodman et al., 2010). Total scores may range from 0 to 20, with higher scores indicating higher difficulties. The SDQ difficulties’ total score comprises a sum of difficulties subscales scores and may vary from 0 to 40, with higher scores representing greater difficulties. The SDQ was adapted to the Lithuanian population and showed good psychometric properties in a previous study (Gintiliene et al., 2004). The internal consistency of the total SDQ difficulties scale in the present study was good (Cronbach’s α = .84), acceptable for Emotional problems (α = .75), Prosocial behaviour (α = .71) subscales, and questionable for Conduct problems (α = .69), Hyperactivity (α = .63) and Peer relationship problems (α = .63) subscales; internal consistency was acceptable for external difficulties (α = .78) and internal difficulties (α = .77) symptom subscales.

*The Child and Adolescent Trauma Screen-Caregiver’s (CATS-C)* versions for 3–6-year-olds and 7–17-year-olds were used (Sachser et al., 2017) to measure potential trauma exposure, probable PTSD, and PTSD symptoms severity. The list of 14 potentially traumatic events (e.g. natural disaster, accident or injury, robbery) and the option to write other events that were not included in the list was given to caregivers before the PTSD symptoms evaluation. Caregivers were asked to provide binary answers (0 = No or 1 = Yes), whether the child had an experience of each potentially traumatic event. The cumulative trauma was calculated by adding all the listed item’s scores.

The CATS-C PTSD evaluation part for 3–6-year-olds comprised 16 items, and for 7–17-year-olds – the 20 items that correspond to all DSM-5 PTSD criteria: Re-experiencing, Avoidance, Negative mood/cognition, and Arousal. Caregivers were asked to think about things that bothered their child over the last two weeks and evaluate each item on a four-point scale (0 = Never; 4 = Almost always). All item scores were added, and the cut-off score for probable PTSD was used as recommended in previous studies: for 3–6-year-olds cut-off score was > =  16 (Nilsson et al., 2021), and for 7–17-year-olds > =  21 (Nilsson et al., 2021). Probable PTSD was calculated only if the child was exposed to a traumatic event. The means of all symptom items were used for comparisons since the CATS-C versions between these age groups differ. Both the CATS-C 3–6 years and 7–17 years versions total items’ internal consistency was excellent (Cronbach’s α = .93 and α = .92, respectively) and good for Re-experiencing (α = .82 and α = .85, respectively), Avoidance (α = .86 and α = .85, respectively) and Negative mood/cognitions (α = .83 and α = .84, respectively) subscales; Arousal subscale was good in 3–6 years version (α = .87) and acceptable in 7–17-years-old version (α = .75).

### 2.3. Data analyses

The internal consistency of scales and subscales was evaluated by calculating Cronbach’s alpha coefficients. To assess the degree of consistency of two independent the OCTS raters, we calculated intraclass correlation coefficients (ICC) based on a single rater, consistency, and 2-way random-effects model. ICC of < .50 was considered poor, .50–.75 moderate, .75–.90 good, and .90–1 excellent (Koo & Li, 2016). The chi-square test was used to analyse the relationship between nominal and categorical variables. Fisher’s exact test was used when one or more cell counts in a 2 × 2 table were less than 5. The significance of the difference in means between two independent groups was calculated using the independent samples t-test. The Kruskal–Wallis H test was used to determine if there were statistically significant differences in the OCTS scores across age groups. If significant differences were identified, post-hoc Dunn’s tests with Bonferroni correction were taken to analyse the differences between specific age groups. In addition, partial correlations between variables while controlling for the child’s age were calculated. IBM SPSS Statistics 29 was used for the analyses. The moderating effects of the study sample (community or risk) on the relationship between the OCTS and SDQ scores (total, internalizing, and externalizing problems) were also tested. Age and gender were included as covariates in the models. For moderation models, we used PROCESS macro v4.2 in SPSS v 29 (Hayes, 2022).

For caregiver-reported SDQ and CATS-C symptom scales, 2.4% of data were missing. For the SDQ scale, 3 cases (=  all missing data) were removed from the analyses that included the respective scale. For the analyses, including the CATS-C, five cases (=  all missing data) were removed. The remaining missing scores were replaced by the mean of the subscale an item represents. It has been shown that for low rates of missing values, individual mean imputation demonstrates good results (Shrive et al., 2006). For one case, all items for the CATS-C avoidance subscale were missing, so this additional case was removed from the analyses, including this subscale or general scale scores.

## 3. Results

### 3.1. Trauma exposure and risk-related characteristics in community and risk subsamples

Based on the caregiver reports about their children, the majority of the total study sample, 76.1% (*n* = 159), were exposed to traumatic events; for risk and community subsamples, the prevalence of traumatic experiences was 86.4% (*n* = 95) and 64.6% (*n* = 64) respectively. The group comparison revealed that experiencing and witnessing violent physical or sexual abuse were significantly more prevalent among children from the risk subsample in comparison to the community subsample (Table 2). The risk subsample experienced significantly more traumatic experiences on average (*M *= 2.73, *SD *= 2.00) in comparison to the community subsample (*M *= 1.22, *SD *= 1.23; *t*(163) = 7.12, *p* < .001) (Table 5). There was a significantly higher prevalence of risk-related demographic characteristics within the risk subsample as well. A bit less than a half (41.0%) of risk subsample children were living with other relatives, guardians, or in the institution; almost half (48.6%) of children’s neither caregiver had higher education, and half of the children (50.5%) received professional psychological services. Caregiver’s alcohol or drug misuse (25.9%) and time in prison (9.3%) were also significantly more prevalent in the risk subsample (Table 1).

**Table 2.** Trauma exposure in community and risk subsamples.

Traumatic experience (CATS-C)	Full sample (*N* = 209)		Community subsample (*n *= 99)		Risk subsample (*n* = 110)		*χ^2^* (1)	*p*	Φ
	*n*	%	*n*	%	*n*	%			
Natural disaster	8	3.9	1	1.0	7	6.4		.070*	
Accident or injury	31	15.1	13	13.5	18	16.5	0.35	.553	0.041
Robbery	0	0.0	0	0.0	0	0.0	-	-	-
Slapped, punched, or beaten up in the family	41	20.0	4	4.2	37	33.9		**<**.001*	
Slapped, punched, or beaten up not in the family	28	13.8	9	9.4	19	17.8	2.99	.084	0.121
Witnessing someone being slapped, punched, or beaten up in the family	47	22.9	7	7.3	40	36.7	24.98	**<**.**001**	0.349
Witnessing someone being slapped, punched, or beaten up not in the family	60	29.3	18	18.8	42	38.5	9.65	.**002**	0.217
Unwanted sexual experience	21	10.4	0	0.0	21	19.8		**<**.001*	
Sexual assault	8	4.0	0	0.0	8	7.5		.007*	
Sudden or violent death of a close one	28	13.6	8	8.3	20	18.2	4.23	.**040**	0.143
Attacked, stabbed, shot or hurt badly	0	0.0	0	0.0	0	0.0	-	-	-
Witnessing someone being attacked, stabbed, shot or hurt badly	7	3.4	0	0.0	7	6.4		.016*	
Stressful or scary medical procedure	87	42.4	41	42.7	46	42.2	0.01	.942	0.005
Being around war	1	0.5	0	0.0	1	0.9		1.000*	
Other events	50	25.9	16	16.7	34	35.1	8.50	.**004**	0.210

Note*.* CATS-C = Child and Adolescent Trauma Screen-Caregiver. Significant differences are presented in bold. *Fisher’s exact test.

### 3.2. The OCTS and other measures scores across gender and age groups

The comparison of the OCTS scores between boys and girls (see online Supplementary Table S1) revealed that, in general, boys scored higher on the total OCTS and all the story stem partial scores. However, only the difference in the Animal story was statistically significant (Boys *M* = 3.69, *SD* = 2.57, Girls *M* = 2.55, *SD* = 2.21; *t*(98) = 2.37, *p* = .020). Additionally, boys demonstrated significantly higher scores in the SDQ for Conduct problems and Hyperactivity while showing lower levels of Prosocial behaviour. There were no significant differences between boys and girls for the CATS-C PTSD symptoms.

We also compared the OCTS scores across five age groups (Table 3). The analysis revealed that the total OCTS score and Stomach ache story’s partial score in the youngest group (3–4 years) were significantly higher as compared to the oldest (8–9 years) children (respectively, *p_adj _*= .006 and *p_adj _*= .009). For the partial score of the Nightmare story, the youngest children had higher average scores than 5-year-old children (*p_adj _*= .001) and 8–9-year-old children (*p_adj _*= .012).

**Table 3.** The OCTS mean comparisons across age groups.

	3–4 years		5 years		6 years		7 years		8–9 years		H (4)	*p*	*E*^2^
	*M (SD)*	*N*	*M (SD)*	*N*	*M (SD)*	*N*	*M (SD)*	*N*	*M (SD)*	*N*			
*OCTS stories*													
Total	4.18 (2.39)^a^	25	2.50 (1.89)^ab^	47	3.00 (2.54)^ab^	47	2.92 (2.39)^ab^	30	2.35 (2.01)^b^	60	12.32	.**015**	0.06
Biking	4.16 (3.12)	25	2.66 (2.74)	47	2.66 (3.06)	47	2.90 (3.39)	30	2.50 (2.71)	60	7.79	.099	0.04
Nightmare	4.44 (3.27)^a^	25	1.87 (2.47)^bc^	47	3.23 (3.26)^abc^	47	3.13 (2.85)^abc^	30	2.18 (2.42)^bc^	60	17.78	.**001**	0.09
Burnt hand	4.88 (3.41)	25	3.30 (2.77)	47	3.06 (3.00)	47	2.97 (2.86)	30	2.75 (2.64)	60	8.56	.073	0.04
Stomach ache	3.92 (3.16)^a^	25	2.23 (2.08)^ab^	47	2.62 (3.02)^ab^	47	2.40 (2.84)^ab^	30	1.82 (2.33)^b^	60	11.39	.**023**	0.05
Animal	3.25 (2.21)	16	2.60 (1.85)	20	3.35 (2.68)	31	3.36 (3.13)	14	3.05 (2.44)	19	0.87	.929	0.01
*SDQ*													
Total	10.44 (5.34)^ab^	25	9.83 (5.66)^a^	47	11.24 (6.99)^ab^	46	14.93 (6.93)^b^	30	13.62 (7.31)^ab^	58	13.90	.**008**	0.07
Conduct problems	2.52 (1.76)	25	1.89 (1.83)	47	2.13 (1.81)	46	2.63 (2.33)	30	2.88 (2.60)	58	5.00	.288	0.02
Emotional problems	2.24 (1.79)^ac^	25	2.04 (2.31)^a^	47	2.65 (2.46)^ac^	46	4.23 (2.45)^b^	30	3.38 (2.55)^bc^	58	19.91	**<**.**001**	0.10
Hyperactivity	4.16 (2.12)	25	3.94 (2.02)	47	4.48 (2.18)	46	5.27 (2.43)	30	4.83 (2.51)	58	6.73	.151	0.03
Peer problems	1.52 (1.39)	25	1.96 (1.67)	47	1.98 (2.12)	46	2.80 (2.28)	30	2.53 (2.26)	58	6.36	.174	0.03
Prosocial behaviour	6.80 (2.33)	25	8.11 (1.76)	47	7.52 (2.27)	46	7.73 (1.89)	30	7.41 (1.95)	58	6.60	.159	0.03
*CATS-C PTSD*													
Total	0.27 (0.38)^a^	24	0.34 (0.49)^a^	47	0.38 (0.50)^ab^	46	0.56 (0.54)^ab^	29	0.58 (0.54)^b^	57	15.77	.**003**	0.08
Re-experiencing	0.17 (0.32)^a^	24	0.28 (0.48)^ab^	47	0.37 (0.52)^ab^	47	0.50 (0.56)^ab^	29	0.51 (0.62)^b^	57	13.47	.**009**	0.07
Avoidance	0.23 (0.47)	24	0.40 (0.80)	47	0.38 (0.64)	46	0.72 (1.01)	29	0.73 (0.86)	57	13.58	.009*	0.07
Negative mood/cognitions	0.21 (0.45)^a^	24	0.24 (0.45)^a^	47	0.31 (0.55)^ab^	47	0.43 (0.58)^ab^	29	0.50 (0.59)^b^	57	16.95	.**002**	0.08
Arousal	0.43 (0.65)	24	0.45 (0.58)	47	0.57 (0.70)	47	0.69 (0.66)	29	0.70 (0.60)	57	10.62	.031*	0.05

Notes*.* OCTS = Odense Child Trauma Screening; SDQ = Strengths and Difficulties Questionnaire; CATS-C = Child and Adolescent Trauma Screen-Caregiver. Significant differences are presented in bold. abc – different letters within the same row indicate significant differences between groups; if two groups share the same letter, this indicates that the difference between the groups was not statistically significant. *The Kruskal-Wallis test showed significant results, but post-hoc pairwise comparisons were insignificant.

### 3.3. OCTS internal consistency and inter-rater reliability of the OCTS

Cronbach’s alpha coefficient for the total OCTS score was excellent, and partial scores were acceptable for all conflict stories, ranging from .76 to .78. For the partial score of the Animal story, Cronbach’s alpha was .66. However, this might be due to a smaller sample size.

In total, 103 of the OCTS interview videos were coded by a second independent rater. Close to half (44.7%; *n *= 46) of double-coded interviews were from the risk subsample. Of all double-coded interviews, 21 included the Animal story. ICC coefficients (Table 4) demonstrated that the degree of consistency between the raters was good for most of the conflict stories, ranging from .72 (Burnt hand story) to .88 (Biking story) except for the Animal story – .70. For the total OCTS score, the ICC coefficient was close to excellent, .76 with the Animal story and without it .89. Again, for the Animal story, the coefficients were moderate, which was likely affected by a smaller sample size.

**Table 4.** Internal consistency and inter-rater reliability across different OCTS stories.

OCTS story	Cronbach’s α (*n*)	ICC [95% CI] (*n*)
Biking	.78 (209)	.88 (103)
Nightmare	.77 (209)	.85 (103)
Burnt hand	.76 (209)	.82 (103)
Stomach ache	.76 (209)	.86 (103)
Animal	.66 (100)	.70 (21)
Total (with Animal)	.92 (100)	.76 (21)
Total (without Animal)	.91 (209)	.89 (82)

Notes*.* OCTS = Odense Child Trauma Screening; ICC = Intraclass correlation coefficient.

### 3.4. Validity of the OCTS in the Lithuanian sample

#### 3.4.1. Multi-method convergent validity of the OCTS

The convergent validity of the OCTS was tested by calculating partial correlations between the main study variables controlling for the child’s age. We found that there were medium, positive correlations between the OCTS and the SDQ total scores, *r* = .34, *n* = 203, *p *< .001, and between the OCTS total and the SDQ external difficulties: Conduct problems, *r* = .35, *n* = 203, *p *< .001 and Hyperactivity, *r* = .30, *n* = 203, *p *< .001. The correlation between the OCTS and the CATS-C total scores was small and significant, *r* = .21, *n* = 200, *p *< .01 (see online Supplementary Table S2).

#### 3.4.2. Discrimination between the risk and community subsamples

The OCTS scores were significantly higher in the risk subsample, with small effect sizes indicated by Cohen’s *d* values ranging from 0.27–0.42. The differences for the Stomach ache and Animal stories were not statistically significant between subsamples. All calculated scores on the SDQ and CATS-C were significantly higher in the risk subsample, with Cohen’s *d* values indicating small to large effect sizes, ranging from 0.42–1.51. Mean comparisons between community and risk subsamples can be found in Table 5.

**Table 5.** Mean comparisons between community and risk groups.

	Full sample		Community subsample		Risk subsample		*t* (*df*)	*p*	Cohen’s *d /* Glass’s Δ [95% CI]
	*M (SD)*	*N*	*M (SD)*	*n*	*M (SD)*	*n*			
*OCTS stories*									
Total	2.83 (2.27)	209	2.37 (2.13)	99	3.25 (2.32)	110	2.85 (207)	.**005**	0.395 [0.120; 0.668]
Biking	2.83 (2.97)	209	2.40 (2.84)	99	3.21 (3.04)	110	1.97 (207)	.**050**	0.273 [0.000; 0.546]
Nightmare	2.76 (2.90)	209	2.15 (2.75)	99	3.30 (2.94)	110	2.92 (207)	.**004**	0.418 [0.139; 0.694]*
Burnt hand	3.23 (2.92)	209	2.60 (2.65)	99	3.80 (3.05)	110	3.03 (207)	.**003**	0.420 [0.145; 0.694]
Stomach ache	2.43 (2.68)	209	2.13 (2.30)	99	2.69 (2.97)	110	1.53 (203)	.127	0.244 [−0.031; 0.517]*
Animal	3.13 (2.45)	100	3.06 (2.41)	47	3.19 (2.51)	53	0.25 (98)	.801	0.051 [−0.342; 0.443]
*SDQ*									
Total	12.03 (6.81)	206	8.86 (5.00)	98	14.91 (6.97)	108	7.20 (194)	**<**.**001**	1.209 [0.885; 1.529]*
Conduct problems	2.41 (2.15)	206	1.43 (1.32)	98	3.30 (2.37)	108	7.08 (171)	**<**.**001**	1.411 [1.071; 1.746]*
Emotional problems	2.90 (2.47)	206	2.23 (2.13)	98	3.50 (2.61)	108	3.82 (202)	**<**.**001**	0.593 [0.306; 0.877]*
Hyperactivity	4.53 (2.29)	206	3.69 (2.00)	98	5.29 (2.29)	108	5.30 (204)	**<**.**001**	0.739 [0.455; 1.021]
Peer problems	2.19 (2.04)	206	1.50 (1.61)	98	2.82 (2.19)	108	4.98 (196)	**<**.**001**	0.824 [0.525; 1.119]*
Prosocial behaviour	7.57 (2.04)	206	8.01 (1.86)	98	7.17 (2.12)	108	3.02 (204)	.**003**	0.422 [0.145; 0.698]
CATS-C cumulative trauma exposure	2.02 (1.85)	206	1.22 (1.23)	96	2.73 (2.00)	110	6.59 (184)	**<.001**	1.224 [0.897; 1.545]*
*CATS-C PTSD*									
Total	0.44 (0.51)	203	0.20 (0.30)	96	0.65 (0.57)	107	7.12 (163)	**<**.**001**	1.508 [1.157; 1.855]*
Re-experiencing	0.38 (0.54)	204	0.18 (0.31)	96	0.56 (0.62)	108	5.61 (162)	**<**.**001**	1.214 [0.887; 1.537]*
Avoidance	0.51 (0.81)	203	0.22 (0.58)	96	0.78 (0.89)	107	5.26 (184)	**<**.**001**	0.944 [0.636; 1.249]*
Negative mood/cognitions	0.35 (0.54)	204	0.11 (0.22)	96	0.57 (0.64)	108	6.93 (134)	**<**.**001**	2.071 [1.666; 2.471]*
Arousal	0.58 (0.64)	204	0.30 (0.42)	96	0.83 (0.69)	108	6.73 (179)	**<**.**001**	1.269 [0.937; 1.595]*

Notes*.* OCTS = Odense Child Trauma Screening; SDQ = Strengths and Difficulties Questionnaire; CATS-C = Child and Adolescent Trauma Screen-Caregiver. Significant differences are presented in bold. * Glass’s Δ is reported in case variances significantly differ between groups (with *SD* of the community group used).

We also compared the OCTS scores between PTSD risk groups based on the CATS-C cut-off scores. In total, 34 (16.7%) children were screened at risk for PTSD. In the community subsample, there were significantly fewer children with probable PTSD (3.1% *vs* 29.0%; *χ^2 ^*= 24.24 (1), *p *< .001). As shown in Table 6, the PTSD risk group had a higher OCTS total score and Nightmare, Burnt hand, and Stomach ache stories partial scores, with small to medium effect sizes *d* = 0.40–0.65.

**Table 6.** Mean comparisons between CATS-C-based PTSD risk groups.

OCTS story	PTSD risk		No PTSD risk		*t* (*df*)	*P*	Hedges’ *g* [95% CI]
	*M (SD)*	*n*	*M (SD)*	*n*			
Total	3.77 (2.33)	34	2.60 (2.12)	169	2.88 (201)	.**004**	0.540 [0.168; 0.910]
Biking	3.35 (2.86)	34	2.71 (2.93)	169	1.17 (201)	.243	0.219 [−0.148; 0.587]
Nightmare	3.71 (3.29)	34	2.54 (2.77)	169	2.16 (201)	.**032**	0.404 [0.034; 0.773]
Burnt hand	4.71 (2.65)	34	2.89 (2.79)	169	3.50 (201)	**<**.**001**	0.654 [0.281; 1.026]
Stomach ache	3.50 (3.12)	34	2.11 (2.32)	169	2.46 (41)	.**018**	0.560 [0.188; 0.930]
Animal	3.75 (2.91)	16	3.00 (2.38)	82	1.11 (96)	.270	0.301 [−0.233; 0.833]

Notes*.* OCTS = Odense Child Trauma Screening; CATS-C = Child and Adolescent Trauma Screen-Caregiver. Significant differences are presented in bold.

We further tested whether the association between the OCTS total and the SDQ total difficulties and between the OCTS total and the SDQ internal and external difficulties depended on the study subsamples. Age and gender were included in the models as covariates. As shown in the online Supplementary Table S3, no significant moderating effects were found, indicating that being in a high-risk vs. community group was not a significant moderator for the OCTS scores (*p**** ***> 0.05).

## 4. Discussion

There is a severe lack of instruments that are constructed to reveal the inner world of young children who might have been traumatized (de Young et al., 2011). The Odense Child Trauma Screening (OCTS) aims to fill the gap in this field by providing a novel measure that combines a story stem methodology with structured play in an attractive and age-appropriate setting for children aged 4–8 years. With the OCTS being a new instrument, there is a need to investigate the psychometric qualities in various samples and the potential to identify signs of traumatization in young children (Alkærsig et al., 2024; Løkkegaard et al., 2021). The current study provides initial data on the OCTS within the Lithuanian context in a community and risk subsamples. It contributes to the existing knowledge on the assessment of traumatization in young children, in particular using the OCTS. In addition to our main aim of the study – to test the reliability and the convergent validity of the OCTS in Lithuania – we also had the opportunity to investigate preliminary data on the prevalence of potentially traumatic events (PTEs) and risk for PTSD in the community and risk subsamples of preschool children in Lithuania, since there is a severe lack of data about these experiences during early childhood. We found that the majority of young children from the community subsample, 64.6%, were exposed to at least one PTE. The most prevalent events were scary medical procedure and physical violence within or outside the family, witnessing it or experiencing it by themselves.

For the reliability analysis we calculated Cronbach’s alpha coefficients according to the OCTS coding manual, where the manifestation of all codes is evaluated for each story separately (Løkkegaard et al., 2018); also following the analyses conducted in the OCTS authors’ original work (Alkærsig et al., 2024). The internal consistency was very good for the total OCTS score. Each of the partial OCTS scores had acceptable internal consistency. All Cronbach’s α’s were above .76, except for the partial score of the Animal story, which had the lowest Cronbach’s α score. For the inter-rater reliability, the intraclass correlation coefficients (ICC) were good, except for the Animal story, which was moderate. In comparison with the data from the Danish results (Alkærsig et al., 2024; Løkkegaard et al., 2021), it seems that OCTS has good inter-rater reliability, even though the scores were somewhat a bit higher in the Danish reports. The results further confirm that the OCTS manualized scoring system can be effectively adapted and applied within the Lithuanian context, and for further reliability analysis of the OCTS it would be essential to explore the internal consistency across the coding system of the OCTS e.g. the five categories of 27 codes or across three categories representing the risk for traumatization.

The comparisons between the community and risk subsamples in the current study indicated that children in the risk subsample generally have higher scores on the OCTS, the SDQ, and the CATS-C measures, reflecting a greater risk for traumatization and mental health difficulties than the community subsample. The OCTS discriminated between the risk and community subsamples for the total score and all the stories, except for the Stomach ache and Animal stories. This could indicate that these two stories, in comparison to others, do not differentiate well between the two groups, or perhaps the results of these two stories can be explained by methodological considerations, such as fewer cases of the Animal story in the total sample or maybe the psychological pressure of the two stories may vary more depending on age. Therefore, the two later stories need further investigation. On the other hand, the Nightmare and Burnt hand stories had significantly higher partial scores for the risk subsample, and the effect size was around 0.4. Comparing the OCTS scores of children identified as at-risk for PTSD and those not at-risk, using caregiver-reported PTSD symptoms via the CATS-C, we found significantly higher scores of the total OCTS score and the partial scores for the Nightmare, Burnt hand, and Stomach ache stories in the PTSD risk group. The OCTS story stems have a different psychological pressure, and some of them might affect traumatized children more intensely (Alkærsig et al., 2024). The results of our analysis indicate that the total OCTS score and the partial scores of the Nightmare, Burnt hand and Stomach ache stories are at the core of identifying the risk for traumatization in young children in Lithuania.

The associations between the total OCTS and the CATS-C were found significant, except for one non-significant association with CATS-C Avoidance symptoms, indicating the initial data of the OCTS to identify young children at risk for PTSD symptoms. Our study corroborates with the previous research, which also found significant correlations between the OCTS and PTSD symptoms based on the Diagnostic Infant and Preschool Assessment (DIPA) in Løkkegaard et al. (2021), except for the PTSD Avoidance symptoms. Furthermore, all associations between the total OCTS score and total SDQ score, as well as all the SDQ difficulties scales scores, were significant and positive. Elevated correlations were found between the total OCTS score and the external difficulties measured with the SDQ Conduct problems and Hyperactivity scales. A similar tendency was found by Alkærsig et al. (2024), where the association between the OCTS total score was significant only with the SDQ total score and with the SDQ Conduct problems and Hyperactivity scales. The OCTS seems to be more sensitive in identifying external mental health difficulties, as reported by caregivers. Moreover, we could test whether the association between the total OCTS score and the SDQ total difficulties as well as internalizing and externalizing difficulties, depended on the study subsamples while adjusting the models for age and gender. We found that there were no significant moderating effects in the group, meaning that the OCTS similarly predicts the SDQ difficulties within both groups and that traumatization signs via the OCTS are similarly related to other difficulties in both groups. The convergent validity analysis of the OCTS calls for further exploration. The OCTS showed the capability to discriminate between the risk and community subsamples, and we found significant associations between the OCTS, SDQ and CATS-C, but on the other hand, the effect sizes of comparisons between groups were small to medium and the significant correlations between the measures were not high.

We explored the total OCTS score and all partial scores between boys and girls and between different age groups. While all scores of the OCTS were elevated for boys, there was only one significant difference between groups for the partial score of the Animal story. This mirrors the tendency in the Danish study (Alkærsig et al., 2024), albeit the difference between Danish boys and girls on the level of total and partial scores did not reach statistical significance. Furthermore, the analysis of the total OCTS scores across the five age groups (3–4, 5, 6, 7 years, and 8–9 years) revealed that younger children (3–4 years) had significantly higher total scores and two partial scores in comparison to older children (8–9 years). Significant differences in partial scores between age groups were found for the Nightmare and Stomach ache stories. Similar findings of elevated the OCTS total scores and partial scores of the Bike story and Stomach ache story for younger children (4-year-olds) in comparison to older children (6–8-year-olds) were found in the preliminary Danish norms (Alkærsig et al., 2024). Our study results indicate that the OCTS has similar effects for boys and girls, but there are age effects, with higher scores for younger children. Therefore, this should be accounted for while evaluating possible traumatization signs. Also, researchers and clinicians should be aware of potential differences in scores for boys and girls that may vary depending on the country of origin.

To sum up, the OCTS shows promising results in a different cultural context than where it was developed, but there should be an awareness of variations in scores between the two countries. Also, small to medium effect sizes of comparisons between groups and low correlations between the measures calls for further exploration of the reliability and validity of the OCTS. Future studies on the OCTS would benefit from cross-cultural examination because we found that the Lithuanian community sample scores were lower than the preliminary Danish norm sample (Alkærsig et al., 2024). Also, the Lithuanian risk sample scores were lower than the Danish risk sample (Løkkegaard et al., 2021). Finally, for the reliability and convergent validity analyses having a more reliable reference standard (e.g. clinical PTSD diagnosis) in future studies could provide useful information on the OCTS validity, including evaluation of possible cut-off points for identifying children at risk. Also, since it is known that in young children the symptoms of traumatization are less specific (Løkkegaard et al., 2019), the coding system of the OCTS is rather broad and covers various aspects. It might be promising to include more age-appropriate self-report measures for young children, and assessing different aspects of traumatization for the OCTS validity testing.

### 4.1. Limitations and future directions

We identified several limitations of our study that need to be addressed. While we aimed to match risk and community subsamples on age and gender, the risk subsample was older. Also, the number of participants varied across different age groups. Therefore, we aimed to control for age in our analysis; however, comparisons between age groups should be interpreted cautiously. On the other hand, there was a similar distribution of girls and boys across groups, and a higher prevalence of risk-related demographic characteristics within the risk subsample allowed us to evaluate the validity of the OCTS. Out of all the OCTS story stems, fever cases were present in the total sample with the Animal story. Therefore, we have considered this when analyzing and interpreting the data whenever possible. Further exploration of the Animal story stem is needed with larger sample sizes.

Also, assigning study participants to risk and community subsamples may have influenced the results. Children were assigned to these groups according to the institutions where the children were interviewed. Therefore, investigation of the OCTS possibilities of targeting traumatized children might have been limited. On the other hand, the data from the study allowed us to distinguish the PTSD risk group according to the CATS-C questionnaire and to test the OCTS possibilities. In future studies, it is recommended to look for methodological solutions to identify risk groups, e.g. based on children’s traumatic experiences. Further studies may also explore and compare the OCTS scoring profiles in risk and non-risk groups and identify the central codes associated with PTSD symptoms in risk and non-risk groups.

We have also observed the limitations related to the study procedure since the data for the risk and community subgroups were collected within different institutions; we could not identify how many caregivers were invited to participate in the study and decided not to. Also, the data from the caregivers was collected on the online platform in the community subgroup, and the paper-pencil method was used in the risk subsample; therefore, there were a few missing data in the caregiver self-reports in the risk subsample. On the other hand, the percentage of missing data was not high, which allowed us to choose the optimal statistical solution for solving this data limitation.

## 5. Conclusions

The present study allowed us to assess the psychometric properties of the Odense Child Trauma Screening (OCTS) in the Lithuanian context, collect data from community and risk subsamples, perform reliability and validity analyses, and enhance the existing knowledge base on the OCTS. The study demonstrated similar psychometric properties of the OCTS in a cultural context different from its original development, although score variations were observed between Lithuanian and Danish samples. The OCTS showed good inter-rater reliability and capability to discriminate between the risk and community subsamples. We also found significant associations between the OCTS, SDQ and CATS-C. On the other hand, the effect sizes of comparisons between groups were small to medium and the significant correlations between the measures were not high. The total OCTS score and the partial scores of the Nightmare, Burnt hand, and Stomach ache stories are relevant in identifying the risk of traumatization in young children in Lithuania.

Future research should focus on further cross-cultural examination, reliability and validity evaluation of the OCTS. In particular, since the coding system of the OCTS is rather broad and covers various aspects, it might be useful to include measures assessing different aspects of traumatization. Also, having a more reliable reference standard (e.g. clinical PTSD diagnosis) could provide useful information on the OCTS validity, including evaluation of possible cut-off points for identifying children at risk. The training, which also includes supervision, is needed for the proper administration of the OCTS; therefore, only trained professionals, such as psychologists, could use the OCTS in professional practice.

## Supplementary Material

Accepted_version_Supplementary_data_2025_02_25.docx

## Data Availability

The datasets of the current study are not publicly available for ethical reasons and data protection.
