# Supplementary material for: Identifying traumatization in young children through structured play: validation of the Odense Child Trauma Screening (OCTS) in Lithuania
Source: Eur J Psychotraumatol. 2025 Mar 10;16(1):2474373. doi: 10.1080/20008066.2025.2474373 (PMC11894742; doi:10.1080/20008066.2025.2474373)
Supplement: Accepted_version_Supplementary_data_2025_02_25.docx [file ZEPT_A_2474373_SM4589.docx]

Table S1. *Mean comparisons between boys and girls*

|  |  | Boys | | Girls | | *t* (*df*) | *p* | Cohen’s *d*  [95% *CI*] |
| --- | --- | --- | --- | --- | --- | --- | --- | --- |
|  |  | *M (SD)* | *n* | *M (SD)* | *n* |  |  |  |
| *OCTS stories* | |  |  |  |  |  |  |  |
|  | Total | 3.17 (2.36) | 86 | 2.59 (2.18) | 123 | 1.81 (207) | .072 | 0.254 [-0.023; 0.531] |
|  | Biking | 3.13 (3.03) | 86 | 2.62 (2.92) | 123 | 1.23 (207) | .222 | 0.172 [-0.104; 0.448] |
|  | Nightmare | 3.19 (3.15) | 86 | 2.46 (2.69) | 123 | 1.80 (207) | .073 | 0.253 [-0.023; 0.530] |
|  | Burnt hand | 3.62 (3.11) | 86 | 2.96 (2.77) | 123 | 1.61 (207) | .110 | 0.226 [-0.051; 0.502] |
|  | Stomach ache | 2.66 (2.73) | 86 | 2.26 (2.64) | 123 | 1.07 (207) | .286 | 0.150 [-0.126; 0.426] |
|  | Animal | 3.69 (2.57) | 51 | 2.55 (2.21) | 49 | 2.37 (98) | **.020** | 0.474 [0.075; 0.870] |
| *SDQ* | |  |  |  |  |  |  |  |
|  | Total | 12.91 (7.07) | 86 | 11.40 (6.58) | 120 | 1.57 (204) | .118 | 0.222 [-0.056; 0.499] |
|  | Conduct problems | 2.77 (2.37) | 86 | 2.15 (1.95) | 120 | 1.98 (160) | **.049** | 0.289 [0.011; 0.567] |
|  | Emotional problems | 2.93 (2.43) | 86 | 2.88 (2.52) | 120 | 0.16 (204) | .875 | 0.022 [-0.255; 0.299] |
|  | Hyperactivity | 4.93 (2.25) | 86 | 4.24 (2.29) | 120 | 2.14 (204) | **.033** | 0.303 [0.024; 0.581] |
|  | Peer problems | 2.28 (2.14) | 86 | 2.13 (1.97) | 120 | 0.54 (203) | .614 | 0.071 [-0.206; 0.348] |
|  | Prosocial behavior | 7.07 (2.15) | 86 | 7.93 (1.89) | 118 | 3.03 (204) | **.003** | 0.428 [0.147; 0.707] |
| CATS-C cumulative trauma exposure | | 2.06 (1.82) | 86 | 2.00 (1.87) | 120 | 0.22 (204) | .824 | 0.031 [-0.246; 0.308] |
| *CATS-C PTSD* | |  |  |  |  |  |  |  |
|  | Total | 0.49 (0.52) | 84 | 0.41 (0.51) | 119 | 1.04 (201) | .298 | 0.149 [-0.131; 0.428] |
|  | Re-experiencing | 0.35 (0.46) | 84 | 0.41 (0.58) | 120 | 0.81 (202) | .422 | 0.114 [-0.165; 0.393] |
|  | Avoidance | 0.58 (0.86) | 84 | 0.47 (0.77) | 119 | 1.01 (201) | .312 | 0.144 [-0.135; 0.424] |
|  | Negative mood/cognitions | 0.39 (0.57) | 84 | 0.32 (0.52) | 120 | 0.88 (202) | .381 | 0.125 [-0.154; 0.404] |
|  | Arousal | 0.66 (0.66) | 84 | 0.52 (0.62) | 120 | 1.58 (202) | .116 | 0.225 [-0.055; 0.502] |

*Notes.* OCTS = Odense Child Trauma Screening; SDQ = Strengths and Difficulties Questionnaire; CATS-C = Child and Adolescent Trauma Screen-Caregiver. Significant differences are presented in bold.

Table S2. *Partial correlations between OCTS, SDQ and CATS scores*

|  |  | 1 | 2 | 3 | 4 | 5 | 6 | 7 | 8 | 9 | 10 | 11 | 12 | 13 | 14 | 15 | 16 |
| --- | --- | --- | --- | --- | --- | --- | --- | --- | --- | --- | --- | --- | --- | --- | --- | --- | --- |
| *OCTS stories* | |  |  |  |  |  |  |  |  |  |  |  |  |  |  |  |  |
|  | 1. Total | - |  |  |  |  |  |  |  |  |  |  |  |  |  |  |  |
|  | 2. Biking | .80*** (206) | - |  |  |  |  |  |  |  |  |  |  |  |  |  |  |
|  | 3. Nightmare | .85*** (206) | .61*** (206) | - |  |  |  |  |  |  |  |  |  |  |  |  |  |
|  | 4. Burnt hand | .80*** (206) | .51*** (206) | .62*** (206) | - |  |  |  |  |  |  |  |  |  |  |  |  |
|  | 5. Stomach ache | .78*** (206) | .49*** (206) | .57*** (206) | .54*** (206) | - |  |  |  |  |  |  |  |  |  |  |  |
|  | 6. Animal | .70*** (97) | .44*** (97) | .55*** (97) | .39*** (97) | .50*** (97) | - |  |  |  |  |  |  |  |  |  |  |
| *SDQ* | |  |  |  |  |  |  |  |  |  |  |  |  |  |  |  |  |
|  | 7. Total | .34*** (203) | .23*** (203) | .27*** (203) | .31*** (203) | .27*** (203) | .29** (97) | - |  |  |  |  |  |  |  |  |  |
|  | 8. Conduct problems | .35*** (203) | .25*** (203) | .28*** (203) | .32*** (203) | .31*** (203) | .20* (97) | .80*** (203) | - |  |  |  |  |  |  |  |  |
|  | 9. Emotional problems | .15* (203) | .08 (203) | .09 (203) | .18** (203) | .08 (203) | .22* (97) | .73*** (203) | .36*** (203) | - |  |  |  |  |  |  |  |
|  | 10. Hyperactivity | .30*** (203) | .21** (203) | .27*** (203) | .23*** (203) | .24*** (203) | .14 (97) | .77*** (203) | .58*** (203) | .38*** (203) | - |  |  |  |  |  |  |
|  | 11. Peer problems | .24*** (203) | .16* (203) | .18* (203) | .19** (203) | .21** (203) | .30** (97) | .72*** (203) | .48*** (203) | .41*** (203) | .33*** (203) | - |  |  |  |  |  |
|  | 12. Prosocial behavior | -.24*** (203) | -.19** (203) | -.20** (203) | -18** (203) | -.20** (203) | -.15 (97) | -.38*** (203) | - .42*** (203) | -.14 (203) | -.28*** (203) | -.34*** (203) | - |  |  |  |  |
| *CATS-C PTSD* | |  |  |  |  |  |  |  |  |  |  |  |  |  |  |  |  |
|  | 13. Total | .21** (200) | .12 (200) | .18* (200) | .21** (200) | .22** (200) | .14 (95) | .60*** (198) | .43*** (198) | .52*** (198) | .46*** (198) | .36*** (198) | -.14* (198) | - |  |  |  |
|  | 14. Re-experiencing | .21** (201) | .15* (201) | .20** (201) | .17* (201) | .20** (201) | .13 (95) | .51*** (199) | .35*** (199) | .45*** (199) | .40*** (199) | .33*** (199) | -.13 (199) | .86*** (200) | - |  |  |
|  | 15. Avoidance | .10 (200) | -.01 (200) | .10 (200) | .14* (200) | .11 (200) | .16 (95) | .33*** (198) | .21** (198) | .33*** (198) | .26*** (198) | .17* (198) | -.01 (198) | .72*** (200) | .56*** (200) | - |  |
|  | 16. Negative mood/cognitions | .23** (201) | .13 (201) | .17* (201) | .25*** (201) | .23*** (201) | .07 (95) | .57*** (199) | .41*** (199) | .49*** (199) | .44*** (199) | .35*** (199) | -.15* (199) | .91*** (200) | .70*** (201) | .60*** (200) | - |
|  | 17. Arousal | .27*** (201) | .19** (201) | .19** (201) | .24*** (201) | .30*** (201) | .15 (95) | .64*** (199) | .49*** (199) | .52*** (199) | .50*** (199) | .42*** (199) | -.26*** (199) | .91*** (200) | .71*** (201) | .52*** (200) | .79*** (201) |

*Notes.* OCTS = Odense Child Trauma Screening; SDQ = Strengths and Difficulties Questionnaire; CATS-C = Child and Adolescent Trauma Screen-Caregiver. All correlations are controlled for the child’s age. Degrees of freedom are presented in brackets.

* *p*<.05; ***p*<.01; ****p*<.001

Table S3. *Moderation analyses of the study group on the relationship between OCTS and SDQ scores*

| Y | X | b | *SE* | *t* | *p* | [95% *CI*] | *R^2^* | *F* (*df*) |
| --- | --- | --- | --- | --- | --- | --- | --- | --- |
| SDQ total | OCTS total | 0.53 | 0.29 | 1.83 | .069 | [-0.04; 1.10] | 0.27 | 14.56 (5, 200), *p*<.001 |
|  | Group | 3.89 | 1.37 | 2.84 | .005 | [1.19; 6.59] |  |  |
|  | OCTS total x group | 0.30 | 0.37 | 0.81 | .421 | [-0.44; 1.04] |  |  |
| SDQ internalizing | OCTS total | 0.21 | 0.17 | 1.18 | .239 | [-0.14; 0.55] | 0.15 | 12.70 (5, 200), *p*<.001 |
|  | Group | 1.66 | 0.83 | 2.01 | .045 | [0.04; 3.29] |  |  |
|  | OCTS total x group | 0.09 | 0.23 | 0.39 | .699 | [-0.36; 0.53] |  |  |
| SDQ externalizing | OCTS total | 0.32 | 0.17 | 1.94 | .054 | [-0.01; 0.65] | 0.28 | 15.53 (5, 200), *p*<.001 |
|  | Group | 2.23 | 0.79 | 2.82 | .005 | [0.67; 3.78] |  |  |
|  | OCTS total x group | 0.21 | 0.22 | 0.99 | .322 | [-0.21; 0.64] |  |  |

*Notes.* OCTS = Odense Child Trauma Screening; SDQ = Strengths and Difficulties Questionnaire. All moderation models were adjusted for age and gender. For the study group, community subsample = 0, risk subsample = 1.
